# Iatrogenic Dural Puncture-induced Abducens Nerve Palsy: Treatment with Epidural Blood Patch

**DOI:** 10.7759/cureus.6622

**Published:** 2020-01-10

**Authors:** Richa Wardhan, Shauna M Wrazidlo

**Affiliations:** 1 Anesthesiology, University of Florida, Gainesville, USA

**Keywords:** dural puncture, post dural puncture headache, abducens nerve palsy, intracranial hypotension, epidural blood patch

## Abstract

Iatrogenic dural puncture (IDP) is a known complication of epidural anesthesia. While operator experience is certainly critical in preventing inadvertent dural punctures, it can happen even in the hands of a skilled operator. Often, IDP presents as post-dural puncture headache (PDPH) without the involvement of cranial nerves; however, infrequent excessive loss of cerebrospinal fluid (CSF) can lead to intracranial hypotension causing compression of the cranial nerves, in particular, the abducens nerve. Here, we describe the case of a patient who suffered from an atypical headache with neurological sequalea from an IDP. Epidural blood patch (EBP) is an effective treatment for PDPH, especially in cases that are not responsive to conservative therapy. However, it may be the first line of defense in patients with neurological symptoms arising from low intracranial hypotension. In our case report, as well as others reported in the literature, there was a complete resolution of the neurological symptoms after the EBP was placed.

## Introduction

Iatrogenic dural puncture (IDP) usually presents as post-dural puncture headache (PDPH) caused by the sudden leak of cerebrospinal fluid (CSF). PDPH is defined as a headache that worsens on attaining upright position and is relieved quickly after resuming recumbent position [1]. It can be frontal or occipital in location, radiating to neck/back/shoulder and is often accompanied by nausea, photophobia, and dizziness. Infrequently, IDP can have an atypical presentation [2]. Here, we describe the case of a patient who developed intracranial hypotension, presenting as diplopia from abducens nerve palsy without the telltale signs of PDPH.

## Case presentation

A 37-year-old female with an oesophageal mass presented for distal oesopago-gastrectomy and roux-en-y oesophagojejunal anastomosis. For the planned operation, a T6-7 epidural was placed by the paramedian approach without complication for post-operative pain control. Ropivicaine 0.2% was started at 8 mL/hr with 4 mL boluses with a once-per-hour allowance. The patient underwent the planned procedure as expected.

On post-operative day one, the patient complained of severe incisional site pain. The epidural catheter was bolused without any relief of pain and sign of a sensory block. After discussion, the patient was offered a replacement of the epidural and she elected to proceed with the plan. There was noted difficulty in placing the epidural via the paramedian approach leading to an unintentional dural puncture on the first attempt. The epidural catheter was then successfully placed at T8-9 by a midline approach with improvement in incisional pain. 

The day following the unintentional dural puncture, the patient complained of new headache, neck, and shoulder pain. The headache was not positional and no other symptoms of a PDPH were noted. The posterior headache along with neck and shoulder pain was described as debilitating. At this point, ultrasound-guided bilateral greater occipital nerve blocks were done with immediate relief of posterior head and neck pain, however, her shoulder pain persisted. 

On post-operative day four, the epidural catheter was removed without any complications. Following the catheter removal, the patient reported cessation of shoulder pain. One week following the unintentional dural puncture, the patient complained of headache and double vision. She described the headache as dull, bilateral but worse on the left, and without a change in the intensity with positional changes. She denied hearing changes and photophobia. She complained of nausea that worsened with sitting or standing. 

**Figure 1:**
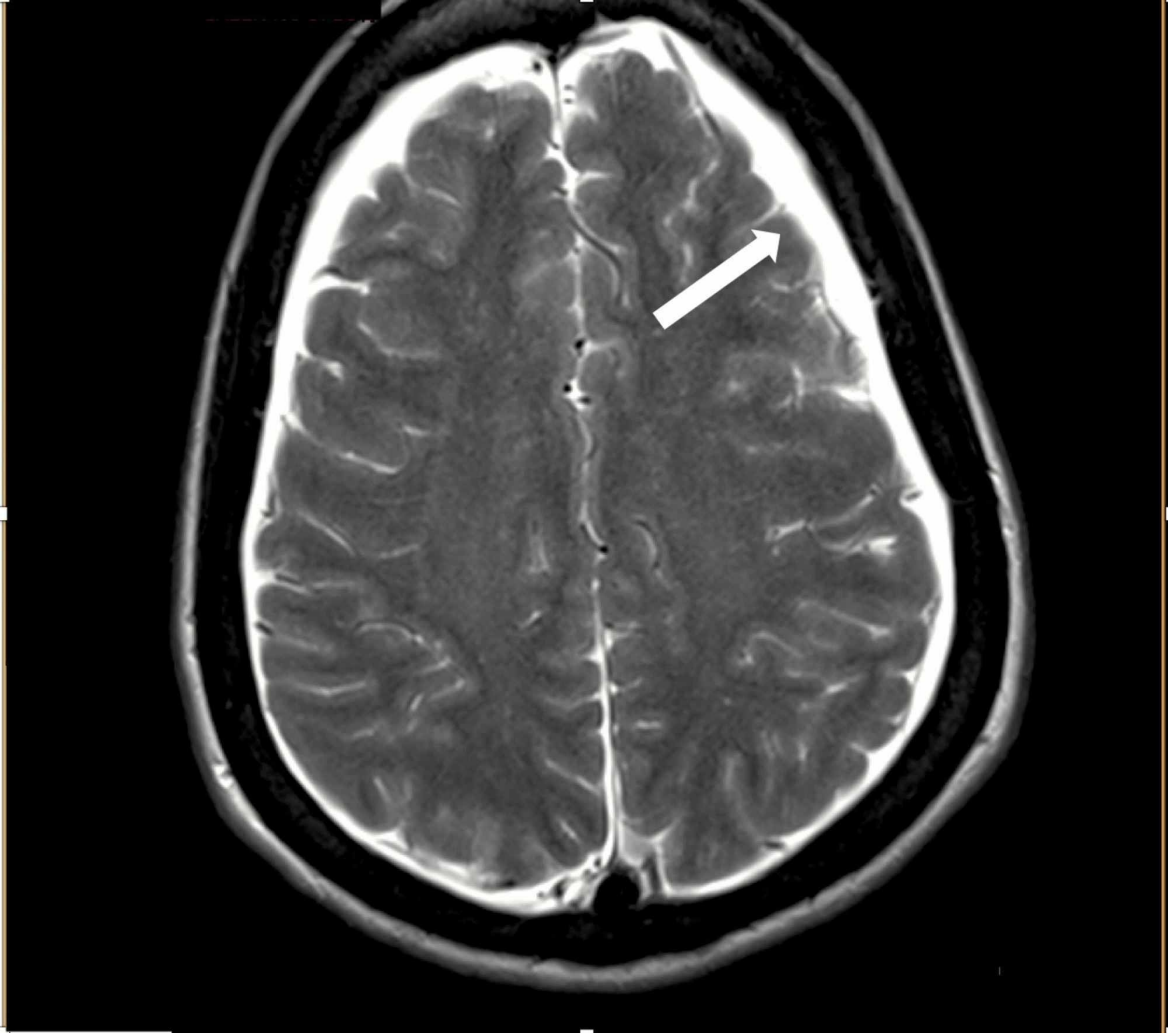
Coronal view magnetic resonance imaging (MRI) depicting subdural effusions (white arrow)

**Figure 2:**
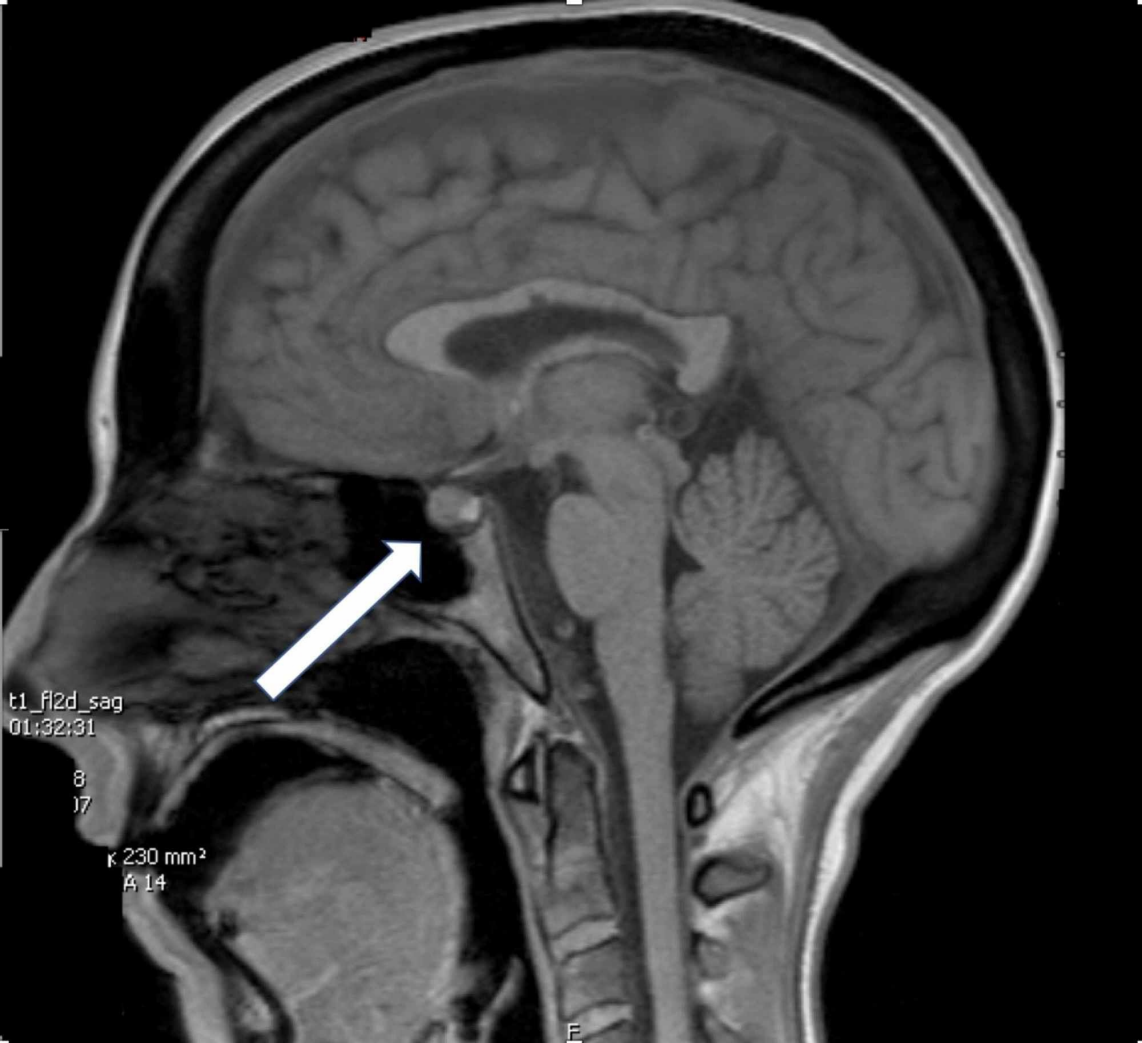
Saggital view of head magnetic resonance imaging (MRI) depicting pituitary bulge (white arrow)

On physical exam, the patient had double vision on rightward gaze (divergence paresis), consistent with right cranial nerve 6 palsy. Intravenous hydration, fiorcet, and caffeine were ordered. Neurology service was consulted and based on their evaluation, concern was raised for microvascular etiology with a low suspicion for PDPH. Computed tomography angiography (CTA) of the head and neck was performed which was negative for carotid or vertebral dissection. In addition, magnetic resonance imaging (MRI) of the brain was performed and findings were consistent with intracranial hypotension (Figures 1-2). Based on these findings, an epidural blood patch (EBP) was suggested. The EBP was placed at the lumbar spine and a total of 15 ml of blood was injected into the epidural space. Following the EBP, the patient’s headache resolved and the cranial nerve palsy improved but persisted. Upon follow-up visit with the surgical services, there were no reported visual complaints.

## Discussion

Abducens nerve palsy presenting as diplopia is a rare but serious complication of intracranial hypotension from CSF leak. Among the cranial nerves causing ophthalmolplegia, the abducens or the sixth cranial nerve is the most frequently involved with horizontal diplopia and blurred vision [3]. Zada et al. further mention that of the patients with cranial nerve palsy secondary to intracranial hypotension, 83% of patients had abducens (VI) nerve palsies, 14% had oculomotor (III) nerve palsies, and 7% had trochlear (IV) nerve paresis [3]. The exact incidence of abducens nerve palsy is unknown but Thomke’s institutional experience put the risk in a range of approximately 1 in 6,000 patients [4]. Abducens nerve palsy due to intracranial hypotension is a benign condition and about 80% of patients recover spontaneously [3].

The abducens nerve is most sensitive to intracranial hypotension. This is not due to its longer course as was earlier believed but due to three acute angulation points between the dural entrance point and its anastomosis with the periarterial sympathetic plexus [5]. The diagnosis of IDP abducens nerve palsy is that of exclusion [2]. Other etiologies including multiple sclerosis, myasthenia gravis, thyroid disease, diabetes, myositis, intracranial tumour, arteriovenous malformation, pachymeningitis, and intracranial hematoma should be ruled out [6-7].

The characteristic MRI findings are typical of intracranial hypotension consisting of small ventricles, diffuse post-contrast meningeal enhancement with downward displacement of the brainstem and subdural effusions. There could also be tearing of small meningeal vessels causing subdural hematoma [8]. Diffuse pachymeningeal enhancement is one of the most common neuroimaging abnormalities in intracranial hypotension which can lead to suspicion of inflammation or neoplasm as the etiology. Clinical correlation is therefore extremely important [6].

Literature search reveals both conservative and invasive therapies [2,8]. Conservative treatment includes intravenous dexamethasone therapy followed by taper and lateral gaze exercises and/or alternate patching of the eyes every two hours for a couple of days followed by Fresnel prism eye lenses. Invasive treatment includes EBP, and in unresolved cases, strabismus eye repair [5].

The timing of an EBP is unclear to derive from the existing literature [8-9]. However, there is an indication that the placement of EBP within 24 hours of presentation of abducens nerve palsy is most effective in treating the palsy by restoring the intracranial pressure back to normal [2]. In a total of 27 patients, in two case reports where patients developed abducens nerve palsy after IDP, 25 patients had complete resolution of abducens nerve palsy from the EBP [3,8].

The mechanism that accounts for the success of EBP is not completely understood. Presumably, when the blood is injected, it enters both the posterior and anterior epidural space [10]. The thecal space is compressed and displaced by the blood. The blood also exits out of the intervertebral foramina and into the paravertebral space. 14 ml of blood can cause a mean spread up to six spinal segments cephalad and three segments caudad. Compression of the thecal space leading to elevation of subarachnoid pressure, may explain the rapid resolution of the headache [10].

## Conclusions

Cranial nerve palsies from intracranial hypotension can occur even in the absence of the classic PDPH. The most common cranial nerve to be affected by intracranial hypotension is cranial nerve VI or abducens nerve. Unilateral abducens nerve palsy is more common than bilateral presentation. In the case of abducens cranial nerve palsy, headaches may precede the palsy, follow it, or maybe completely absent. This knowledge prevents unnecessary and dangerous workups including a diagnostic lumbar puncture. Cranial nerve palsies can present days after IDP clouding the diagnosis. Gadolinium-enhanced MRI is the investigation of choice in clinching the diagnosis of cranial nerve palsies following IDP. While there are no clear recommendations for when to place an EBP for treating the neurological symptoms, earlier treatment with EBP has a better prognosis.
